# Novel 2019 coronavirus: Genome structure, clinical trials, and outstanding questions

**DOI:** 10.1177/1535370220920540

**Published:** 2020-04-19

**Authors:** Manasi P Jogalekar, Anurag Veerabathini, Prakash Gangadaran

**Affiliations:** 1 Brigham and Women’s Hospital, Harvard Medical School, Boston, MA 02115, USA; 2 Maxim Integrated Products Inc., Chandler, AZ 85225, USA; 3 Department of Nuclear Medicine, School of Medicine, Kyungpook National University, Kyungpook National University Hospital, Daegu 41944, Republic of Korea; 4 BK21 Plus KNU Biomedical Convergence Program, Department of Biomedical Science, School of Medicine, Kyungpook National University, Daegu 41944, Republic of Korea

**Keywords:** SARS-CoV-2, COVID-19, coronavirus, Wuhan, 2019-nCoV, MERS-CoV, SARS-CoV, China

## Abstract

Novel 2019 coronavirus has created havoc across the globe since its emergence in December 2019 in Wuhan, China, and fast spreading potential. While we were able to identify the causative agent within a few days of the disease outbreak, several questions still remain unanswered. In this review, we discuss the extent of virus spread, current statistics, SARS-CoV-2 genome organization, comparison between the novel coronavirus and causative agents involved in previous outbreaks, ongoing clinical trials and myths associated with the virus. Lastly, we provide insights into the future perspectives which could prove useful for the scientific community as they work on finding the cure against the disease.

**Impact statement:**

Early availability of the sequence, the genetic material of SARS-CoV-2 (the virus that causes COVID-19), has prompted efforts towards identifying a safe and effective vaccine in the current public health emergency. To that end, understanding the pathophysiology of disease is crucial for scientists around the world. Since conventional vaccine development and manufacturing may take several years, it is important to think about alternative strategies that we could use to mitigate imminent catastrophe. We hope that this article will open up new avenues and provide insights that could potentially save hundreds of lives affected by COVID-19.

## The beginning

A deadly coronavirus infection originated in China in late December 2019, when a group of people visiting China Seafood Market in Wuhan developed an unusual respiratory syndrome. By the time Chinese authorities announced the outbreak, the disease had already spread among several people.^1^ Unfortunately, the Chinese doctor who suspected the possibility of Middle East respiratory syndrome (MERS)-like disease outbreak has died due to exposure to the very same virus. The infection spread globally within a short time due to extensive international travel to celebrate Chinese Lunar New Year. Scientists worldwide have now established that the mysterious illness is coronavirus disease 2019 (COVID-19) and the virus associated with it is identified as novel severe acute respiratory syndrome coronavirus 2 (SARS-CoV-2) or also referred to as 2019-nCoV.^2^

COVID-19 is characterized by symptoms such as fever, sore throat, lesions in lungs, and difficulty in breathing.^3^ People have also experienced dry cough, lymphopenia, fatigue, anorexia, arrhythmia, and shock.^4^ The severity of symptoms depends on the overall health of a person, extent of spread and virulence of the strain. A study involving infected people in Wuhan showed a tad higher incidence in males (54.3%) than females.^4^ While children are equally prone to getting infected as adults, the severity of symptoms appears to be less in children compared to adults.^5,6^ However, researchers are still gathering more evidence to back this observation.

## Current statistics and global situation

As of 28 March 2020, 649,904 confirmed cases have been found globally, out of which 81,999 cases are in China. The number of confirmed cases and deaths in Hubei Province alone has gone up to 67,801 and 3177, respectively. The cumulative number of deaths in Mainland China has reached 3299. Apart from China, 567,905 cases and 26,950 deaths have been confirmed in 199 countries so far; the United States (Cases: 115,547; deaths: 1891), Italy (Cases: 92,472; deaths: 10,023) and Spain (Cases: 72,248; deaths: 5812) being the worst-hit countries outside China.^7^ Although, 137,283 out of the total number of infected people worldwide recovered, it has recently been indicated that they may have reduced lung function on a long-term basis and this aspect of COVID-19 warrants further investigation.^7,8^ Most of the reported cases are likely due to being in contact with the affected individual, knowingly (to care for their loved one) or unknowingly (by being admitted to the hospital where COVID-19 patients were being treated, to receive treatment for other ailments or by being around an asymptomatic person).^9^ Interestingly, reports indicate that the people who had no apparent connection with China Seafood Market have also been infected, confirming human-to-human transmission.^10^ It is likely that there are more cases than the above-mentioned numbers, since the people with mild sickness may self-quarantine, and thereby go unreported. The Center for Systems Science and Engineering (CSSE) at Johns Hopkins University has developed a user-friendly and an interactive web-based tool to enable research community and public health officials to track COVID-19 cases in real-time.^7^ Bespoke Inc. has developed a Chatbot called “Bebot” using artificial intelligence tools that keeps people updated with the latest information on the coronavirus and suggests preventive measures to take to avoid infection.^11^

World Health Organization (WHO) has declared global health emergency and has identified COVID-19 as a pandemic.^12,13^ In an effort to contain COVID-19 outbreak, travel restrictions and lockdown of cities within China have been imposed. Other countries are also enforcing similar restrictions on domestic and international travel, with increased scrutiny being conducted for passengers arriving from China. As a result, COVID-19 cases in China are going down, while those in other countries are rising. Health care workers and medical doctors are working overtime and on weekends to treat existing and new cases that are being added to the COVID-19 patient pool each day. It is of utmost importance that we do our share to help mitigate this global pandemic. SARS-CoV-2 being a respiratory virus, its transmission could occur within six feet through droplets released via infected person’s coughing and sneezing.^10^ A striking new study has indicated that SARS-CoV-2 can stay in the air up to 3 h after being aerosolized and anywhere from 4 h to three days on surfaces.^14^ There are a few basic precautions that each one of us can and should take. Simple things like washing hands thoroughly before and after meals as well as after using toilet, avoiding touching face (eyes, nose, and mouth) with hands, sanitizing doorknobs or other surfaces before touching, and covering the nose while sneezing and coughing, can go a long way. It is always a better idea to self-quarantine for a few days if you are sick rather than going to work and putting others at risk. Similarly, it is also important to stay away from people who are sick and advise them to get tested.

## SARS-CoV-2 genome structure

2019-nCoV (a group 2B CoV) genome has been successfully sequenced using a sample isolated from a positive patient, a noteworthy accomplishment in itself.^15,16^ Molecular tests developed using this data have been tremendously helpful in determining the accurate number of COVID-19 cases. Homology analysis revealed that both SARS-CoV-2 and SARS-CoV have ∼73% similar receptor binding domains with 8 out of 14 amino acid residues conserved in SARS-CoV-2.^15^

**Figure 1.**
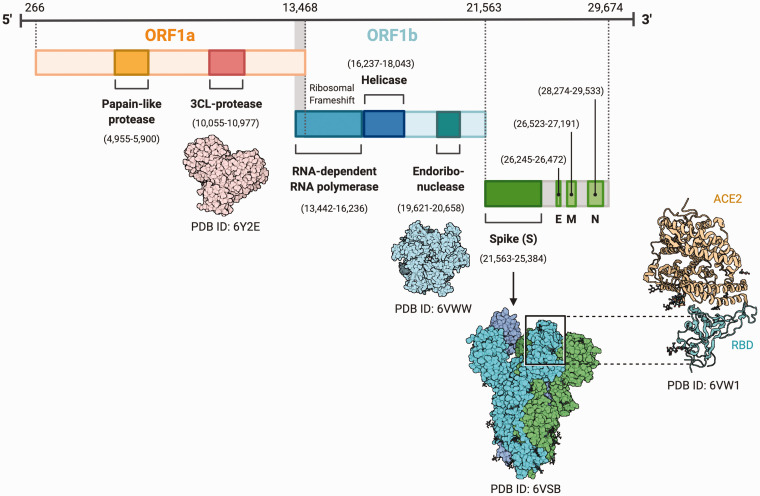
Genomic organization of SARS-CoV-2.^15^ SARS-CoV-2 genome consists of ∼30 Kb RNA strand. It is organized as follows: 5ʹ end, Papain like protease, 3C‑like serine protease (3CL-protease; PDB ID: 6Y2E), RNA-dependent RNA polymerase, Helicase, Endoribonuclease (PDB ID: 6VWW), Spike protein (PDB ID: 6VSB) containing receptor-binding domain (RBD; PDB ID: 6VW1) which binds to a human receptor—angiotensin converting enzyme 2 (ACE2), envelope protein (E), membrane protein (M), and nucleocapsid protein (N), 3ʹ end. Enzymes are essential for viral replication and spike protein is needed for host cell entry. *Created with BioRender. (A color version of this figure is available in the online journal.)

Like other coronaviruses, SARS-CoV-2 genome is made up of a single-stranded RNA, about ∼30 kb in size (Figure 1). The genome encodes for both structural and non-structural proteins. Structural proteins include spike glycoprotein (S; consists of 2 domains—S1 and S2), envelope protein (E), membrane protein (M), and nucleocapsid protein (N), all situated near 3ʹ end of the strand.^17^ Lu *et al.*^15^ suggests that S protein binds to angiotensin-converting enzyme 2 (ACE2) receptor in humans with high affinity via its receptor-binding domain (RBD) to facilitate host cell entry. SARS-CoV-2 S-protein is also capable of binding ACE2 receptors from bat, civets, and swine.^18^ Enzymes such as papain‑like cysteine protease and 3C‑like serine protease (3CL-protease) are located near 5ʹ end of the open reading frame and play a role in the proteolytic breakdown of polyprotein 1a (pp1a) and pp1ab to form non-structural proteins such as RNA‑dependent RNA polymerase, helicase, and endoribonuclease. These proteins enable viral replication and transcription. Crystal structures of spike protein and various enzymes encoded by SARS-CoV-2 genome have been deposited in protein data bank (PDB).^19–22^

## Comparison of SARS-CoV-2 with severe acute respiratory syndrome coronavirus (SARS-CoV) and Middle East respiratory syndrome coronavirus (MERS-CoV)

Due to technological advances, SARS-CoV-2 genome was sequenced within a month of an outbreak, while it took several months to identify causative agents in previous outbreaks as SARS- and MERS-CoV.

Phylogenetically, SARS-CoV-2 has shown the closest association with a couple of bat coronaviruses, bat-SL-CoVZC45, and bat-SL-CoVZXC21 (88%), then with SARS-CoV (79%), and lastly with MERS-CoV (50%).^15^ RaTG3, another bat CoV sequence, has shown 92% sequence identity with SARS-CoV-2.^18^ S protein and protein 13 of SARS-CoV-2 have shown the lowest sequence identity (80% and 73% respectively) with other group 2B CoVs, compared to other proteins (>90% sequence identity).^15^ It has been suggested that furin, a host cell enzyme found in small intestines, lungs and liver, activates a site on the SARS-CoV-2 S protein, justifying rapid worldwide spreading of COVID-19 infection and symptoms observed in COVID-19 patients.^23^ S protein encoded by SARS-CoV-2 was also considerably longer than other viruses.^15^ Epidemiological data suggest that the estimated transmission rate of SARS-CoV-2 is similar to that of SARS- and MERS-CoV.^24^ Yet, SARS-CoV (10% mortality) and MERS-CoV (37% mortality) have been found to be more lethal than SARS-CoV-2 (∼2% fatality).^9,25^ Like MERS-CoV, SARS-CoV-2 has an incubation period of 5.2 days on average and death of the infected patient occurs within a median duration of 14 days from the onset of symptoms, with this duration being 11.5 days in elderly people ≥70 years of age.^26,27^

Coronaviruses are zoonotic in nature, which means that they have the ability to transmit between animals and humans. It is likely that SARS-CoV-2 may also have had intermediate animal host before it was transmitted to humans, similar to SARS-CoV and MERS-CoV.^15^ SARS-CoV-2 has been found to infect people of all ages including individuals older than 50 as well as anyone having underlying conditions such as cardiovascular disease, respiratory ailments, and diabetes. Similar trends were observed in previous outbreaks caused by SARS- and MERS-CoV.^28,29^

Rothe *et al.*^30^ reported that asymptomatic patients are also capable of transmitting SARS-CoV-2; a similar phenomenon was observed in case of MERS-CoV but not in SARS-CoV.^31^ Initial reports suggest that SARS-CoV-2 is able to spread via healthcare workers just like its counterparts, indicating the possibility of super spreading.^31,32^ Super spreading events can be triggered by host factors^33^ and sometimes even via animals.^34^ Although all three viruses maintain low R0 (the number of people infected by a single patient), sudden rapid increase in the number of SAR-CoV-2 cases suggest super spreading events at play.

In an effort to understand viral transmission, serial interval and incubation period for SARS-CoV-2 were determined. Results suggest shorter serial interval periods (∼5–6 days) as well as viral shedding one to two days prior to the onset of symptoms, indicating possible pre-symptomatic transmission of SARS-CoV-2.^35^ What’s more, a science news article reported the presence of viral RNA in blood, stool, sputum, and urine of patients long after the end of symptoms, indicating multiple routes of transmission.^36^

## Current efforts to develop intervention and clinical trials

Coronavirus research is moving forward at record speed. Currently, there is no cure for 2019-nCoV infection. However, government agencies, global scientific community, public health officials, and medical doctors have come together in an unprecedented fashion to tackle the coronavirus problem from all angles. A quick search of Clinicaltrials.gov with keywords “COVID-19” and “Novel 2019 Coronavirus” shows ∼66 ongoing trials in various phases studying various aspects of COVID-19 and screening potential drug candidates.^37^

Within weeks of the outbreak, pharmaceutical companies Co-Diagnostics and Novacyt developed molecular tests that specifically recognize 2019 coronavirus.^38,39^ A pharmaceutical giant, Gilead and a biotech startup, Moderna therapeutics, have developed a small molecule antiviral called remdesivir and novel mRNA vaccine respectively, against 2019-nCoV. Remdesivir has previously been used to treat Ebola- and MERS-CoV-infected patients. Since MERS-CoV exhibits similarities to SARS-CoV-2, the drug molecule is being tested in COVID-19 patients.^40^ The results for clinical trials NCT04280705, NCT04292730, and NCT04292899 are highly anticipated.^41^ In addition, chloroquine, a drug routinely used in the clinic to treat malaria, has shown success in inhibiting 2019-CoV replication *in vitro.*^27^ On the contrary, Moderna has employed a novel strategy for their vaccine by delivering chemically synthesized mRNA encoding instructions for the S protein using lipid nanoparticles (NCT04283461).^40^ Inovio Pharmaceuticals Inc. is developing and testing a DNA-based vaccine in preclinical models.^41^ Companies like Sanofi and Janssen are working on cell-based vaccines.^40^ Regeneron pharmaceuticals and Vir Biotechnology are developing neutralizing monoclonal antibodies, the approach that worked against Ebola, SARS-CoV, and MERS-CoV infections, and testing in preclinical models.^40,41^ Several clinical trials are exploring the potential of interferon alpha and beta (IFN-α and IFN-β) as well as anti-retroviral drugs such as Lopinavir/Ritonavir (normally used to treat HIV) to fight COVID-19.

Recent study demonstrates that SARS-CoV-2 uses ACE2 for host cell entry and is dependent upon TMPRSS2, the serine protease, for S protein priming, indicating that using TMPRSS2 inhibitors could be an effective intervention strategy to block viral entry into the host.^42^ The same human receptor was used by SARS-CoV to facilitate entry into the lower respiratory tract epithelial cells.^43^ As discussed previously, blockade of ACE2 receptor and furin enzyme may prove beneficial in preventing viral entry into the host cells.

Another approach that has shown promise in China is the use of a technique called plasmapheresis, where plasma rich in antibodies against SARS-CoV-2 is taken from recovered patients and injected into sick patients. It has been observed that COVID-19 patients had better blood oxygenation and reduced inflammation, 24 h post-injection of antibodies. In the United States, this approach is being implemented by the Mount Sinai Health System in New York.^44^

Most of the clinical trials mentioned above are going to take ∼3–6 months to complete phase I and another ∼6–8 months for phase II before they can get an approval. In the meantime, we will have to rely on supportive care to treat COVID-19 patients by administering antipyretics for fever, expectorants for cough, oxygen therapy for severe dyspnea, antibiotics for sepsis, and Glucocorticoids (adults) or methylprednisolone (children) to mitigate body’s immune response.^4,12^

## Distinguishing facts from myths

With the overwhelming amount of new information being shared each day, it is easy to miss the fine line between facts and untrue stories or baseless claims. For example, the claim that hot baths can prevent us from getting COVID-19 is not true. Similarly, cold temperatures, hand dryers, ultraviolet lamps, and antibiotics cannot kill SARS-CoV-2. No matter what the external temperatures are, human body temperature always remains constant (37°C). Likewise, we do not have any information to validate the claims that garlic, chlorine baths, or pneumonia vaccine are effective against SARS-CoV-2. In addition, there is no evidence to substantiate the claim that SARS-CoV-2 spreads via mosquitoes.^45^ Unfortunately, panic situations created by such myths have forced the general public to stock up on supplies even though they are not at risk, thereby resulting in disruption of supply chains, crashing of stock markets, price surging, and shortage of essential supplies like gloves, masks, and goggles, putting health care workers at risk across the globe.^45,46^ To meet an increasing demand, governments and industries have been asked to up their manufacturing by 40%.^46^ To avoid this situation from happening again, we request our readers to validate the information using trusted sources before sharing it on social media platforms.

## Outstanding questions and future perspectives

While the scientific community all over the world is racing to find a cure for 2019-nCoV infection, some key questions still remain unanswered. The sources of primary COVID-19 infection and its subsequent transmission as well as involvement of any intermediate animal hosts are still unclear. In addition, there is a lot of uncertainty regarding the pathophysiological mechanisms of SARS-CoV-2 advent. The sooner we find answers to these pressing questions, the better chance we would have at stopping the spread of COVID-19 infection. We have some promising leads in terms of finding the source of primary infection and the transmission of the virus. For example, one study has reported the involvement of two different viral strains in COVID-19 outbreak.^47^ Recent reports also suggest human-to-human transmission of the virus as well as the possibility of transmission via snakes^48^ or bats^15^ but further experiments are needed to confirm this information. There is an urgent need to generate animal models of COVID-19 in order to determine *in vivo* response to SARS-CoV-2. While both SARS-CoV and MERS-CoV were able to infect non-human primates, the potential of SARS-CoV-2 to infect other species is still unknown.

While it is remarkable that several pharmaceutical companies are developing vaccines and antivirals for COVID-19, the potential drug candidates will have to go through various regulatory channels before getting approval. Once the vaccine formulations are approved, companies will need to manufacture the doses in large amounts to meet increasing global demand. To ensure that the entire vaccine development process goes smoothly, large funds are needed. The Bill and Melinda Gates Foundation recently launched COVID-19 Therapeutics Accelerator in collaboration with Wellcome and Mastercard to help identify treatments for COVID-19. The joint venture is backed by a generous funding of $125 million.^49^ Bill Gates, in his recently published the New England Journal of Medicine article, urges national governments as well as private organizations to support low- and middle-income countries (LMICs), usually the hardest-hit population in such situation.^50^ Since the process of vaccine development takes a lot of time, it may be a better idea to screen currently existing drugs for their ability to treat COVID-19. That way, we may be able to find a solution quickly and in a cost-effective manner. In addition, each country should implement emergency action plan to manage a pandemic situation like COVID-19. A good example of this would be Taiwan’s disaster management plan, which they employed during initial stages of the outbreak. As a result, they were able to significantly contain the virus early and currently have a very few cases (283), despite the country’s close proximity to China.^51^ Lastly, we should develop or take advantage of existing artificial intelligence (AI)-powered tools to quickly screen compound libraries to identify potential antiviral candidates. Various hospitals in China and South Korea are currently using machine learning algorithms to scan thousands of CT images for signs of COVID-19,^51,52^ an approach that will reduce the burden on healthcare workers, increase disease surveillance, predict disease hotspots, and speed up COVID-19 diagnosis, which is exactly what we need at this crucial hour.
